# Emergency medical services providers’ perspectives on the use of artificial intelligence in prehospital identification of stroke- a qualitative study in Norway and Sweden

**DOI:** 10.1186/s12873-025-01300-1

**Published:** 2025-07-28

**Authors:** Ann-Chatrin Linqvist Leonardsen, Camilla Hardeland, Andreas Dehre, Glenn Larsson

**Affiliations:** 1 Østfold University College/Østfold Hospital Trust, Postal Box Code 700, Halden, 1757 Norway; 2 Østfold University College, Postal Box Code 700, Halden, 1757 Norway; 3 PICTA, Prehospital Innovation Arena, Lindholmen Science Park, Gothenburg, Sweden; 4 PreHospen – Centre for Prehospital Research, University of Borås, Borås, Sweden

**Keywords:** Ambulance, Artificial intelligence, Emergency medical services, Emergency medicine, Feasibility, Paramedic, Prehospital care, Stroke

## Abstract

**Background:**

Stroke is a large and increasing health challenge, leading to acquired physical disability and mortality. A rapid diagnostic assessment in the acute phase of a stroke is crucial and highly time dependent. Studies suggest that artificial intelligence (AI) could contribute for prognostication, prediction and resource optimization in suspected stroke cases in prehospital emergency care. The objective of the current study was to explore Emergency Medical Services providers’ perspectives on using AI in the prehospital assessment of patients with a suspected stroke diagnosis.

**Methods:**

A qualitative study design following stroke case simulation with an AI-based diagnostic tool was used. One focus group and ten dyadic interviews were conducted comprising 24 participants from three ambulance stations in Norway and Sweden respectively. Data were analyzed following Braun and Clarke’s steps for thematic analysis.

**Results:**

Three themes were identified, namely (1) Another tool in the toolkit, (2) Trust is essential, and (3) The devil is in the details. The participants underlined that the AI-based tool was just an addition to their usual assessment, including symptoms, anamnesis, and vital parameters, as well as their own ‘clinical eye’. Moreover, trust was needed from various stakeholders for the tool to have a function in the patient pathway. Finally, size and weight, as well as the ability to differentiate between hemorrhagic and thrombotic stroke were central aspects for the tool to be feasible.

**Conclusion:**

Emergency Medical Services providers mainly rely on their own clinical eye, combining symptoms, anamnesis and measurement of vital parameters when assessing suspected stroke patients. AI-based tools may be used as support in the decision-making process, however this depends on the establishment of trust in the tool across EMS providers, neurologists and other health professionals.

## Background

Globally, stroke represents a significant and escalating health challenge, contributing to acquired physical disabilities and ranking as the second leading cause of mortality in middle- to high-income nations [1]. Over 101 million individuals currently live with the aftermath of stroke, with approximately 67% of these individuals being under the age of 70. Risk factors associated with stroke include elevated systolic blood pressure, hyperlipidemia, high body mass index, elevated fasting blood glucose levels, exposure to ambient particulate matter pollution, atrial fibrillation, and smoking [2, 3]. Among incidents of stroke, ischemic strokes account for over 62% of cases, while intracerebral hemorrhages constitute approximately 28% [1]. The Trial of Org 10,172 in Acute Stroke Treatment (TOAST) classification system is the most prevalently employed mechanism-based subclassification system for cerebral ischemia, categorizing strokes into five subtypes: [1] large artery atherosclerosis [2], cardioembolic [3], small vessel occlusion [4], stroke of other determined etiology, and [5] stroke of undetermined etiology [4]. Hemorrhagic strokes may manifest as primary intraparenchymal hemorrhages or subarachnoid hemorrhages (SAH).A rapid diagnostic assessment in the acute phase of an acute stroke is crucial and highly time dependent [5, 6]. After an ischemic stroke, the goal of treatment is to restore blood flow to the affected area within the first hours after the onset of stroke symptoms. The main very early treatments for ischemic stroke include intravenous thrombolytic therapy (tissue plasminogen activator or Tenecteplase) or mechanical thrombectomy [7]. The treatment of a hemorrhagic stroke depends upon the cause of the bleeding (e.g., high blood pressure, use of anticoagulant medications, head trauma, blood vessel malformation). The initial care includes: (1) determining the cause of the bleeding, (2) controlling the blood pressure, (3) stopping any medication that could increase bleeding, (4) measuring and controlling the pressure within the brain and within the skull. A ventriculostomy can also be used to drain blood that has collected in the brain as a result of the stroke [8]. To exclude intracerebral hemorrhage and large irreversible infarctions, neuroradiology is mandatory using either cerebral computer-tomography (CT) or Magnetic Resonance Imaging (MRI) [9, 10].

Current guidelines advocate for training Emergency Medical Services (EMS) providers in recognizing stroke symptoms and notifying the hospital in advance of patient arrival [11]. Despite this recommendation, there is no consensus on the optimal organization of such training [12]. Although hospitals globally utilize the National Institutes of Health Stroke Scale (NIHSS) in acute stroke situations, agreement is lacking on the most suitable stroke scale for use in prehospital settings [12–15]. A recent randomized controlled trial (RCT) conducted in Norway [12] aimed to ascertain whether EMS providers employing the NIHSS in ambulances might improve communication with hospitals, refine triage processes, and enhance the diagnostic precision of acute stroke cases. Findings indicated that the NIHSS increased the identification of patients with low NIHSS scores and subtle symptoms prehospital. However, it did not enhance diagnostic accuracy.

Several initiatives have been introduced aiming to reduce symptom onset to treatment time in acute stroke. These include streamlining in-hospital logistics [16–18], public information campaigns [19, 20], and developing prehospital pre alert systems [21, 22]. Studies suggest that artificial intelligence (AI) in stroke can be used to provide image interpretation and generate estimations of patient outcomes. It can also be useful for centers without dedicated stroke specialists [23, 24]. Several studies also suggest a promising future for AI for prognostication, demand prediction for ambulance services and resource optimization in prehospital emergency care. However, most studies are retrospective, and the need for prospective validation of AI tools before implementation in clinical settings is emphasized [25].

The objective of the current study was to explore EMS provider’s perspectives on using a tool based on AI algorithms in the prehospital assessment of patients with a suspected stroke diagnosis.

## Methods

### Design

The study had a qualitative design, using either dyadic or focus group interviews to get in-depth information about the participants’ experiences after simulating a stroke case using a CE marked AI-based portable tool that uses microwaves for detection of stroke [25]. Both focus groups and dyadic interviews allow participants to interact in response to open-ended research questions [26, 27]. This approach was both pragmatic and appropriate, due to making the researchers (two ambulance nurses, one with a PhD, both male, one female nurse anesthetist/PhD/Professor and one female nurse/PhD) able to concentrate on a clearly defined topic (AI in prehospital assessment of stroke) [26]. The choice of either dyadic or focus group was made of pragmatic reasons, and what was feasible for the participants.

The study is reported in-line with the COREQ (Consolidated criteria for Reporting Qualitative research) Checklist [28].

### Setting

The project was conducted in Norway and Sweden, respectively. In Norway, the healthcare regions are responsible for hospitals and specialized healthcare, 519 car ambulances, nine planes and 13 helicopters, 43 ambulance boats and 16 emergency medical dispatch centers (EMDs). Regular ambulances are mainly operated by two emergency medical technicians (educated at vocational school level), nurses or paramedics (educated at a bachelor’s degree level) [29].

In Sweden, medical healthcare in hospitals and primary care, including emergency care services, are administered by county councils. There are approximately 820 car ambulances, nine helicopters and five aircrafts (for long distance medical transports). The ambulances are mainly staffed with a specialized nurse (registered nurse with a postgraduate education in prehospital emergency care) and a nurse or a nurse assistant (specialist training in prehospital emergency care). There are some examples of vehicles staffed with medical doctors or specially trained nurses, but without possibilities to transport patients. There are also examples of ambulances purely for non-acute transports [30]. In Norway, 9158 cerebral strokes were registered in 2021 [31]. Of these, seven out of ten patients had one or more of the symptoms: fascial paresis, paresis in one arm or leg, speaking difficulties. In Sweden, the numbers in 2021 were 27 000 strokes registered in 25 400 persons [32].

### Sample

A purposive sampling method was used. An invitation to participate was sent to managers of all ambulance regions in a county in Norway with 320 000 inhabitants, and three regions in Sweden with approximately 2,300 000 inhabitants. Inclusion criteria were EMS providers working clinically, experienced with handling of stroke patients. In total, 18 EMS providers from three different regions were included in Sweden, and six EMS providers from three different regions in Norway.

### Simulations

Participants were invited to participate in simulations as basis for the interview. Two different, standardized, patient cases were presented where stroke symptoms of varying degrees could be noted were developed based on actual situations from the ambulance, by ambulance nurses specially trained in simulation development and facilitation. The cases were reviewed by the research team to ensure that they were realistic and appropriate for the study objective. The simulations were facilitated by two of the researchers experienced with working as ambulance nurses and simulation (AD and GL) and conducted in an appropriate location at the ambulance stations, using equipment from the ambulances, similar in both countries.

Before the simulation, participants were instructed in how to use the tool based on microwave technology and AI-algorithms for detection of haemorrhagic or ischemic stroke. The device was designed like a helmet to be used on a hospital bed or ambulance stretcher. The participants were informed that the tool/device could be applied if and when they felt appropriate, and that patients needed to lay still throughout the duration of the measurement (approximately 45 s). Participants simulated both cases in pairs.

### Data collection

An interview guide was developed based on relevant theory [5, 12, 33] and on discussion between the research team members. The guide focused on aspects such as how the participants experienced using the AI-based tool, whether they trusted the result, and their thoughts about implementing such tool in their assessment of suspected stroke patients (see Box 1 Interview guide).

**Box 1:** Interview guide

1. How did you experience using the tool in simulation. Positive aspects? Negative aspects? 2. Did you trust the result? 3. In clinical practice, what would you weigh the most: the recommendation from such tool or your own assessment/other tools?
4. What are your thoughts regarding implementation of another tool in the assessment of stroke patients? 5. What are your thoughts regarding user-friendliness? (Weight? Size?) 6. What are the potential consequences for clinical practice if using such tool?

In Sweden and in one of the Norwegian cases, participants were interviewed dyadic, in the same pair as in the simulation. Additionally, in Norway, two pairs (four participants) were interviewed together. The interviews were information rich, and saturation, defined as no identification of further themes in consecutive interviews, was assumed met after these interviews [34, 35]. The interviews in Sweden were conducted by two of the researchers (AD and GL), while all researchers were present in the Norwegian interviews (ACLL, CH, AD and GL) after agreement with the participants.

The interviews were audio recorded using University of Oslo’s digital application “Nettskjema Diktafon”, which enables secure collection and storage of data, as well as automatic transcription.

### Analysis

Data were analysed using inductive, thematic analysis as recommended by Braun & Clarke [36]. The analysis followed five steps. In step 1, three of the researchers (ACLL, CH and AD) validated the transcripts through listening to the audio recordings and revising the transcripts accordingly. As such, this also represented familiarization with the data. In step 2, the first author (ACLL) coded the data inductively, through highlighting aspects of the data that represented any meaningful in relation to the study aim. These codes were reviewed by another researcher (AD). Then, in step 3, the first author (ACLL) combined codes across interviews to preliminary themes, by assessing codes representing the same topic or aspect of interest. These themes were reviewed, discussed and revised by all authors (ACLL, CH, AD and GL) in step 4. In step 5, the themes were reviewed against the complete data set and the study aim, determining the significance of the themes. Table 1 provides an example of the analytic steps.

**Table 1:** Example of the analytic steps

Transcript	Codes	Preliminary themes	Theme
And symptoms.You get a collated picture of it and try to investigate whether it is a stroke or any suspicion of stroke. Even if you don’t exclude one or the other you would consider the most time critical…A mixed compot of anamnesis, symptoms and some vital parameters…This may be useful in the diffuse cases, where we don’t really know, and a low suspicion…In those cases we could use it to exclude stroke…(Sweden, interview 3)	Symptoms Collated picture Investigate Stroke or not Suspicion Exclude Consider Time critical Mixed compot Anamnesis, symptoms and vital parameters May be useful in diffuse cases Don’t really know Exclude stroke	A collated picture Stroke is time critical Useful in diffuse cases	Another tool in the toolkit

### Ethics and approvals

The project was based on the principles stated in the Declaration of Helsinki [37]. All participants provided their informed, signed, written consent to participate. The Norwegian knowledge sectors’ service suppliers assessed the project (project no. 952723), ensuring that the planned processing of data was in line with the legislations. No ethical approval was required in either Norway or Sweden, as the study did not involve patients and fell outside the scope of the Norwegian regulations [38] and the Swedish Ethical Review Act.

## Results

In total, 24 EMS providers, of which 17 females, participated in 11 interviews across three regions in Sweden and Norway respectively. In Sweden, all interviews were conducted dyadic, while in Norway one was conducted dyadic and one as a focus group interview with four participants. The participants’ median age was 38.5 years (range 27–58 years), and their median years of experience from working in the ambulance was 8.5 years (range 8 months-28 years). Only two of the Norwegian participants were also nurses, while all the Swedish participants were nurses. The interviews lasted from 20 to 54 min. In Norway, all three regions had recently implemented an application for neurologic scoring in stroke suspected cases, however few were familiar with using this. In Sweden, one of the regions had implemented such application some years ago that was actively in use.

Through analysis, three themes were identified, namely (1) Another tool in the toolkit, (2) Trust is essential, and (3) The devil is in the details. Quotes are marked as S (Sweden) or N (Norway) and interview number.

### Another tool in the toolkit

All the participants across interviews and countries stated that an AI-based tool could be useful in the assessment of patients with suspected stroke. However, whether they would actually use the tool or not varied both between regions, participants and patient cases. All the participants reported that their first action in patients with suspected stroke was to assess the patient’s symptoms, and whether the patient had any paralysis, difficulties speaking or other visible signs. Several of the participants across interviews and countries also stated to use their “clinical glance”, as stated in S2:

I’ve worked so long, and seen so many patients…The clinical glance, and the feeling you get almost immediately when you see the patient…it’s really the largest cornerstone…Then, you measure more parameters to either confirm or deny this feeling.

As such, in some of the interviews participants reported that this “glance” made the basis for their decision alone, especially in critical cases or at locations nearby a hospital. Here, several of the participants reported not using any time for further assessment of the patient, referring to a “load and go” strategy to get as fast as possible to the hospital. In such situations, they reported that they would not use any time to add any tool, AI-based or not, that took time to apply. In their opinion, their experience and clinical judgement alone could detect an ongoing stroke and the need for hospital treatment. Still, this led to challenges in those cases where treatment location was depending on whether it was a thrombotic or hemorrhagic stroke.

In locations where they had implemented the NIHSS or a stroke application, these were applied in parallel with measurements of vital parameter. Then, participants would continue with the anamnesis and ask either the patient or relatives about previous incidents and/or comorbidities, depending on the patient’s condition. One of the participants said:

It’s very broad in the beginning. You may have many differential diagnoses, and while examining the patient you pick away issues. (S3)

As such, all the participants across interviews and countries stated that an AI-based tool would be most effective in cases with more subtle clinical symptoms, such as headache or dizziness. This was described e.g. in S5:

It’s those patients with diffuse symptoms. The ones with a dropping corner of the mouth, with aphasia and hemiplegia. They may not win a lot….

Here, and AI-based tool was described to be “another tool in the toolkit”, “a piece in a puzzle”, a “part of the compote” or “a complement”. Similar input was provided in all the interviews, underlining the challenges in the assessment of stroke without established symptoms and many potential differential diagnoses. However, most of the participants stated that they would weigh the result from the AI-based tool least, as stated in N2:

I think I emphasize the tool least…It will be a decision based on everything, age, symptoms… And even if we may have an explanation, I always confer the physician (in hospital) once too much than too little.

### Trust is essential

When discussing their assessment and decision-making as ambulance workers, several of the participants emphasized perceiving lack of trust, especially from hospital physicians. This was exemplified in N2, where one of the participants referred to a recent experience:

Two weeks ago, I had a patient who was paralyzed in the right side 13 years ago. Now, he had a dropping corner of the mouth at the left side and was confused. The neurologist did not trust that at all, but after a lot of discussion we were allowed to come in. The day after, I checked up on the patient, and he was completely paralyzed on the left side. We have struggled with that they don’t trust us. Even if we stand there, they don’t believe the symptoms, even if they’re completely obvious.

The same perception was reported in several of the other interviews in both Norway and Sweden, whether it was lacking trust from the primary care center or the hospital physicians. Several of the participants across interviews underlined the importance that the whole chain of care had to know about the AI-tool to build trust between links in the chain. One of the participants in S1 said: ”They (the physicians) sit in there. In their reality. We may be right, but they still want to do their own assessment. This may lead to friction from time to time”.

This was a two-way issue, also relating to when “the tool” indicated negative results (no stroke). A participant in S7 said:

It feels like something the physicians will be scratching their head about…But, if this is implemented and the physicians start to trust it, maybe the dizziness-patients may be referred to primary care to a larger extent.

Moreover, it was underlined that the EMS providers themselves needed to trust the tool. This seemed to differ slightly between Norway and Sweden. In Norway, all the participants across interviews and regions stated that “I must choose to trust the tool”. However, in Sweden several of the participants expressed doubts about whether they should trust the results from an AI-tool. For example, a participant in S2 prompted:

If the tool says no, no stroke, and all I see is stroke, it will be…It may be similar to trusting an ECG that seems ok, but you have an ash grey, extremely ill patient in front of you.

In all the Swedish interviews this skepticism was present, and most of them concluded that if the AI-tool reported “no stroke”, they would not feel confident to transport them elsewhere than the hospital. Finally, some of the participants also mentioned that the patients would need to trust the tool to feel satisfied with a “no stroke” result (S8).

### The devil is in the details

Regarding details of an AI-based tool to use in suspected stroke cases, all the participants across interviews and countries agreed on most aspects. First, all agreed that it was essential that the tool was able to differentiate between hemorrhagic and thrombotic stroke. All the participants underlined that the main focus when handling patients with suspected stroke was time, and to work as efficient as possible to not “steal time from the patient”. Being able to decide on either thrombolysis or thrombectomy would be essential, especially in regions where different hospitals provided different treatments. Transporting a patient for thrombectomy could in some regions mean a transport time of up to two hours, while thrombolysis could be performed in a hospital nearby. A participant in S5 described it like this: “It is valuable time for the patient. Today, we actually measure time to thrombolysis or thrombectomy”.

As such, being able to differ between haemorrhage and thrombosis was assumed making it easier to require a helicopter.

Second, all the participants agreed that they avoid using various tools in public, and that they prefer to examine the patient inside the vehicle. This was both due to being able to assess the patient in a calm environment, and to avoid exposure of the patient. Then, the size of the tool was assumed of importance. The ambulance was assumed as “overloaded” already, and few of the participants perceived to have any room for another tool. They all underlined already having too many bags to bring to the patient, and consequently, further equipment was assumed redundant.

The participants across regions and countries disagreed on whether all ambulances should be equipped with the AI-based tool, just some or whether it should be located at the ambulance station. This was related to whether it was feasible to fit the tool into the ambulance, how frequent they assumed to need it, and the geographical distances in the ambulance station’s catchment area.

Finally, the Norwegian participants underlined that the AI-based tool needed to be included in their guidelines as well as anchored in management, to indicate when it should be used, in which patients, and which implications the results should provide.

## Discussion

To our knowledge, this is the first study EMS provider’s perspectives on using an AI-based tool in the assessment of suspected stroke patients. EMS providers perceived that an AI-based tool could be useful to support their own assessment, but mainly as an addition. Moreover, they emphasized the importance of building trust in such new tool both in physicians, other health personnel, in themselves and in patients, for being used. Finally, aspects such as size, weight, and the ability to differ between hemorrhagic and thrombotic stroke were seen central for the tool to be feasible in the assessment of stroke patients.

All participants indicated that their initial action upon encountering a patient with suspected stroke was to evaluate the patient’s symptoms, specifically assessing for paralysis, speech difficulties, or other visible manifestations of stroke [39–41]. This evaluation was followed by measuring vital signs and gathering patient history from either the patient, hospital records, or relatives. Subsequently, additional tools, including AI-based applications, were employed. Previous studies suggest that prehospital assessment, informed by initial symptoms and actions such as the Face, Arms, Speech Test (FAST), cannulation, performing electrocardiograms, neurological evaluations, and obtaining information from family members or bystanders at the scene, can increase the duration of on-scene time [39–41]. Delays in prehospital stroke care pose a significant obstacle to successful treatment due to the narrow therapeutic window for thrombolytic interventions, which are most effective when administered shortly after stroke onset [42]. Internationally, multiple initiatives and tools have been explored or adopted to reduce on-scene time, including the deployment of mobile stroke units equipped with specialized personnel and computed tomography (CT) scanning capabilities [43–45]. However, the expense associated with these units may limit their widespread adoption. Additional strategies involve the use of prehospital intracranial ultrasound to examine intracranial arteries [46], telecommunication technologies to connect with neurologists [47, 48], and the utilization of the National Institutes of Health Stroke Scale (NIHSS) via mobile applications [12]. None of these strategies have been systematically implemented at the national level in Norway or Sweden.

Moreover, participants stated that an AI-based tool could be most feasible in patients with diffuse symptoms. The diversity of stroke presentations and hereby difficulties in the assessment of suspected stroke cases, have been underlined in several studies [49–51]. Hence, the diagnosis of stroke is not always straightforward, and similar symptoms may develop in a number of medical conditions. The issue is not only to initiate appropriate treatment in acute suspected stroke, but also to avoid inappropriate use of expensive and potentially harmful medications [51, 52].

In the current study, participants emphasized that for the AI-based tool to be used, both EMS providers, neurologists, other healthcare professionals and the patients needed to trust the tool as part of the assessment. A 2025 systematic review found that trust in digital healthcare is complex and can influence digital healthcare use, adoption, acceptance, and usefulness [53]. The authors state that patients’ trust in digital healthcare interventions, including AI, is impacted by issues such as human interaction, perceived risks, privacy concerns, data accuracy, quality of the intervention, digital literacy, satisfaction, experience, or level of education and income. Healthcare professionals’ trust is developed by observing how the tools perform, and whether it optimizes the services. In stroke assessment, this would imply supporting the decision to rapidly initiate intravenous thrombolytic therapy or to transport the patient to a hospital with mechanical thrombectomy or skull drainage opportunities.

EMS providers in the current study perceived that the size and weight of the AI-based tool were appropriate. The tool was reported to be easy to use, and not time consuming. However, all the participants reflected on whether the ambulance could accommodate further equipment, and whether they themselves could carry any more “bags”. Ambulances need to be durable yet lightweight to ensure maneuverability and fuel efficiency. Inside the vehicle, another challenge is securing the medical staff and patient during transport [54]. Any decision support tools must be validated and applied appropriately, and clear guidelines are needed to define how useful systems are in clinical practice [23].

### Limitations

In-line with the qualitative design lies the limited generalizability of our findings. Also, our findings may not be transferable to healthcare systems with different prehospital care models or resource constraints. This study was conducted in three regions in Norway and Sweden respectively, and it may be argued whether the results are transferable to other settings and countries.

There is professional heterogeneity between Norwegian and Swedish participants which may represent a bias in the analysis. However, the credibility and trustworthiness of our findings are supported by the transparent reporting of the analysis, also including information about whether the quoted participants were Norwegian or Swedish. The research team consists of both ambulance nurses, nurses and a nurse anesthetist.

It may be seen a limitation that the researchers performing the simulations also performed the interviews in Sweden and participated as observers in the interviews in Norway. As such, we could have strengthened the dependability of our findings through either inviting participants to review the analysis and results, or through inviting researchers without the same preconception.

## Conclusion

EMS providers mainly rely on their own clinical eye, combining symptoms, anamnesis and measurement of vital parameters when assessing suspected stroke patients. AI-based tools may be used as support in the decision-making process, however this depends on the establishment of trust in the tool across EMS providers, neurologists and other health professionals. Further studies are needed to assess the effectiveness and feasibility of such tools in stroke management.

### Implications for practice

The findings suggest that EMS providers primarily depend on their clinical judgment, integrating observed symptoms, patient history, and vital sign measurements when evaluating patients suspected of having a stroke. To advance the utility of AI in stroke management, future research is necessary to evaluate the effectiveness and feasibility of these technologies in clinical settings. This research could facilitate the development of protocols that incorporate AI tools, thereby potentially enhancing diagnostic accuracy and improving patient outcomes. Additionally, training programs aimed at familiarizing healthcare providers with AI applications could further support the integration and acceptance of these tools in routine stroke care.

## Data Availability

Data is available upon reasonable request to the first author.

## Declarations

### Use of artificial intelligence

The authors did not use a generative artificial intelligence (AI) tool or service to assist with preparation or editing of this work. The authors take full responsibility for the content of this publication. GPT4 Omni was used to proof-read the manuscript after peer review.
